# Supplementary material for: Effect of Exposure to Particulate Matter on the Ocular Surface in an Experimental Allergic Eye Disease Mouse Model
Source: Bioengineering (Basel). 2024 May 16;11(5):498. doi: 10.3390/bioengineering11050498 (PMC11118833; doi:10.3390/bioengineering11050498)
Supplement: Supplementary file 1 [file bioengineering-11-00498-s001.zip › bioengineering-2975236-supplementary.pdf]

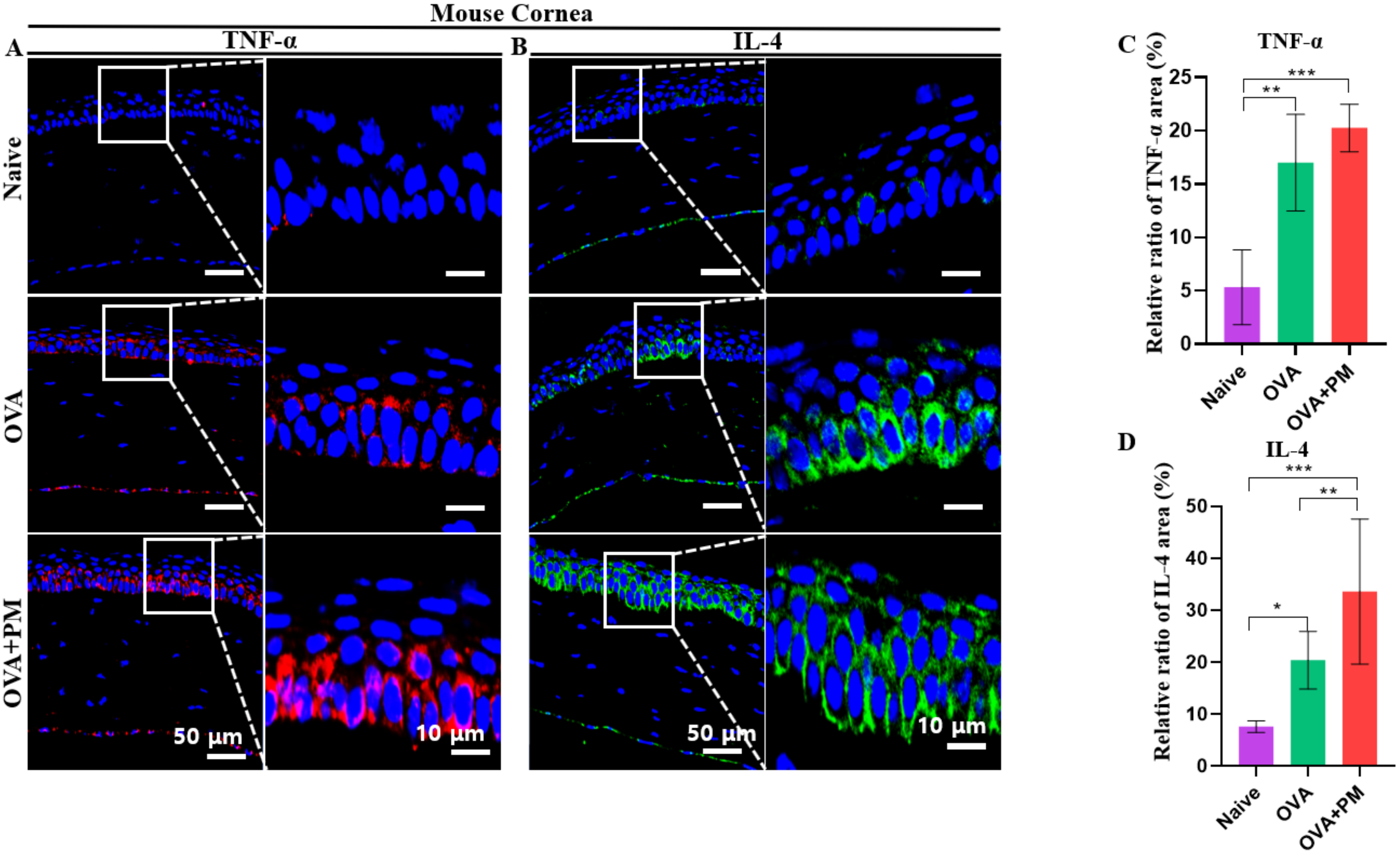

Figure S1. PM exposure triggers allergic anti-inflammatory cytokines infiltration in an AED mouse model. (**A**) Immunofluorescence staining for TNF-α in cornea in naive, OVA and OVA+PM exposed groups. (**B**) Immunofluorescence staining for IL-4 in cornea in naive, OVA and OVA+PM exposed groups. (**C**) Changes in the positive area of TNF- α in cornea. (**D**) Changes in the positive area of IL-4 in cornea. White rectangles indicate TNF-α and IL-4 in cornea; shown in higher-power fields. The scale bar is indicated by a white bar in the lower right corner. In (**C** and **D**) data are presented with bar graph with mean ± SEM ($n = 5$, * $p < 0.05$, ** $p < 0.01$, *** $p < 0.001$, significant difference by one-way ANOVA followed by the Tukey test).

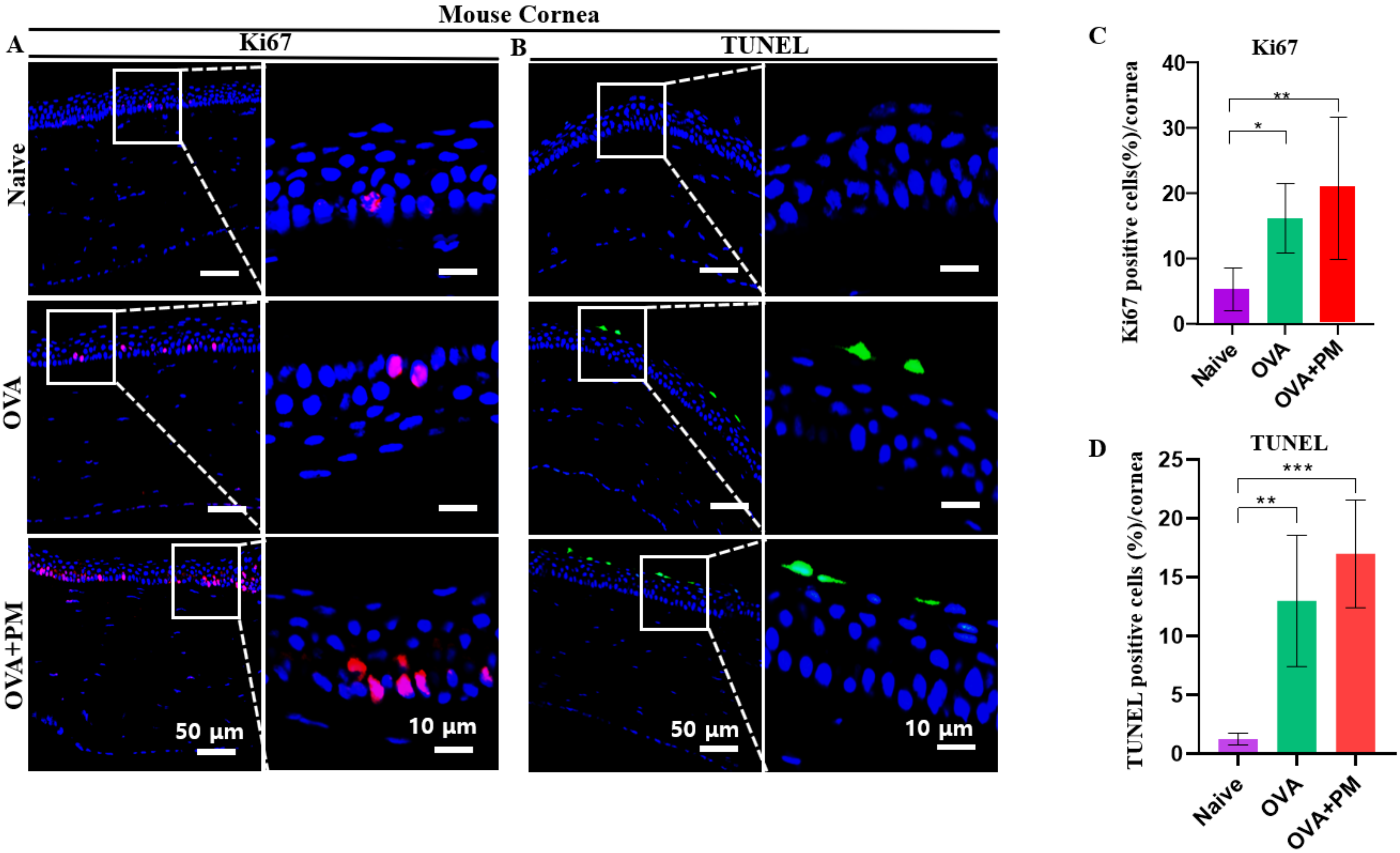

Figure S2. Exposure of PM effects on elevated cell proliferation and cellular apoptosis in an AED mouse model. (**A**) Immunofluorescence staining for Ki67 in cornea in naive, OVA and OVA+PM exposed groups. (**B**) TUNEL staining for cellular apoptosis in cornea in naive, OVA and OVA+PM exposed groups. (**C**) Changes in the ki67 positive cells in cornea. (**D**) Changes in the TUNEL positive cells percentage in cornea. White rectangles indicate the ki67 and TUNEL positive cells cornea showing in higher-power fields. White rectangles indicate the ki67 and TUNEL positive cells in cornea**;** shown in higher-power fields. The scale bar is indicated by a white bar in the lower right corner. In (**C** and **D**) data are presented with bar graph with mean ± SEM ($n = 5$, * $p < 0.05$, ** $p < 0.01$, *** $p < 0.001$, significant difference by one-way ANOVA followed by the Tukey test).
